# Graphitic Carbon Nitride with Dopant Induced Charge Localization for Enhanced Photoreduction of CO_2_ to CH_4_

**DOI:** 10.1002/advs.201900796

**Published:** 2019-07-26

**Authors:** Junwei Fu, Kang Liu, Kexin Jiang, Huangjingwei Li, Pengda An, Wenzhang Li, Ning Zhang, Hongmei Li, Xiaowen Xu, Haiqing Zhou, Dongsheng Tang, Xiaoming Wang, Xiaoqing Qiu, Min Liu

**Affiliations:** ^1^ School of Physics and Electronics Central South University Changsha 410083 Hunan P. R. China; ^2^ College of Chemistry and Chemical Engineering Central South University Changsha 410083 Hunan P. R. China; ^3^ School of Materials Science and Engineering Central South University Changsha 410083 Hunan P. R. China; ^4^ School of Physics and Electronics Hunan Normal University Changsha 410081 Hunan P. R. China; ^5^ Department of Chemistry and Key Laboratory for Preparation and Application of Ordered Structural Materials of Guangdong Province Shantou University Shantou 515063 Guangdong P. R. China; ^6^ State Key Laboratory of Powder Metallurgy Central South University Changsha 410083 Hunan P. R. China

**Keywords:** CO_2_ photoreduction, dopant, excitation orbit direction, graphitic carbon nitride, intrinsic charge localization

## Abstract

The photoreduction of CO_2_ to hydrocarbon products has attracted much attention because it provides an avenue to directly synthesize value‐added carbon‐based fuels and feedstocks using solar energy. Among various photocatalysts, graphitic carbon nitride (g‐C_3_N_4_) has emerged as an attractive metal‐free visible‐light photocatalyst due to its advantages of earth‐abundance, nontoxicity, and stability. Unfortunately, its photocatalytic efficiency is seriously limited by charge carriers′ ready recombination and their low reaction dynamics. Modifying the local electronic structure of g‐C_3_N_4_ is predicted to be an efficient way to improve the charge transfer and reaction efficiency. Here, boron (B) is doped into the large cavity between adjacent tri‐s‐triazine units via coordination with two‐coordinated N atoms. Theoretical calculations prove that the new electron excitation from N (2p*_x_*, 2p*_y_*) to B (2p*_x_*, 2p*_y_*) with the same orbital direction in B‐doped g‐C_3_N_4_ is much easier than N (2p*_x_*, 2p*_y_*) to C 2p*_z_* in pure g‐C_3_N_4_, and improves the charge transfer and localization, and thus the reaction dynamics. Moreover, B atoms doping changes the adsorption of CO (intermediate), and can act as active sites for CH_4_ production. As a result, the optimal sample of 1%B/g‐C_3_N_4_ exhibits better selectivity for CH_4_ with ≈32 times higher yield than that of pure g‐C_3_N_4_.

Photocatalytic reduction of CO_2_ to hydrocarbon products has been considered as a promising way to achieve the recycling use of CO_2_ in atmosphere by using solar energy.^1^ Since Wang et al. first reported the photocatalyst of g‐C_3_N_4_ in 2009,^2^ g‐C_3_N_4_ has become a star in water splitting^3^, ^4^, ^5^, ^6^, ^7^ and CO_2_ reduction.^8^, ^9^, ^10^ Although many studies have been widely carried out, its CO_2_ reduction performance is still far from the actual application requirements and the product is mainly CO (2‐electron reduction product),^11^, ^12^ due to the high recombination rate of charge carriers and low reaction dynamics.^13^, ^14^, ^15^, ^16^

It is well known that electrons are generally excited from N atoms to C atoms in g‐C_3_N_4_. Moreover, the electrons are mainly localized around N, especially the two‐coordinated N atoms, which have been considered as the active sites for photocatalytic reaction.^17^ The localized electrons on N atoms indicate that electrons are hard to transfer from N atoms to C atoms. Furthermore, the excited electrons (from N atoms to C atoms) need to re‐transfer from C atoms to the N atoms (active sites) for catalytic reaction, which bring the high recombination rate of charge carriers and the low reaction efficiency of g‐C_3_N_4_.^10^, ^18^ We therefore take the view that introducing modifier elements—atoms that could tune the electron excitation, transfer and localization in g‐C_3_N_4_—would contribute to the improvement of charge transfer, separation, and reaction dynamics.^19^

Herein, boron (B) atoms were selected to build a good affinity with N atoms in the large cavity between adjacent tri‐s‐triazine units.^20^, ^21^ Theoretical calculations shows that electron excitation from N (2p*_x_*, 2p*_y_*) to B (2p*_x_*, 2p*_y_*) in B‐doped g‐C_3_N_4_ is much easier than N (2p*_x_*, 2p*_y_*) to C 2p*_z_* in pure g‐C_3_N_4_ due to the same orbital direction (Figures S1 and S2, Supporting Information), improved the intrinsic charge transfer, localization and thus the reaction dynamics.^22^, ^23^, ^24^ As a result, the B‐doped g‐C_3_N_4_ showed a higher yield for 8‐electron involved reduction product of CH_4_, which is about 32 times that of the pure g‐C_3_N_4_. We also consider the other nonmetal doped g‐C_3_N_4_, such S and P doped g‐C_3_N_4_. The project density of states (PDOS) of S and P doped g‐C_3_N_4_ was shown in Figures S3 and S4 (Supporting Information). We found obvious S dopant states near the valence band (VB) for S‐doped g‐C_3_N_4_, which is formed by the hybridization of N 2p*_z_* and S 3p*_z_*. Similarly, a dopant level of P lies between the VB and the Fermi level in P‐doped g‐C_3_N_4_ system. Compared with S and P doping, the dopant level of B is higher and locates near the Fermi level. The electronic localization functions (ELF) of S‐doped g‐C_3_N_4_ and P‐doped g‐C_3_N_4_ (Figure S5, Supporting Information) show that the dopant nonmetal element can also induce the same charge localization. This work provides a new strategy to tune local electronic structure property of g‐C_3_N_4_ for boosting photocatalytic performance.

Density functional theory (DFT) studies establish B doping as a promising candidate to modify g‐C_3_N_4_ in light of its good charge localization (**Figure** **1**). The density of states (DOS) of pure g‐C_3_N_4_ and B‐doped g‐C_3_N_4_ obtained from DFT calculations are given in **Figure** **2**.^21^, ^25^, ^26^, ^27^, ^28^ The valence band of pure g‐C_3_N_4_ is mainly composed by N 2p orbitals, and the conduction band (CB) is mainly contributed by C 2p orbitals (Figure 2a).^2^ When the pure g‐C_3_N_4_ was excited by light with appropriate wavelength, the electrons in the N 2p orbitals will jump into the C 2p orbitals. Differently, the CB of B‐doped g‐C_3_N_4_ is composed by C 2p and B 2p orbitals (Figure 2d). The DOS of C 2p, N 2p, and B 2p with three different orbital directions (*x*, *y*, *z*) are provided for comparison. From Figure 2b,c, the top of VB and the bottom of CB in pure g‐C_3_N_4_ are mainly contributed by N (2p*_x_*, 2p*_y_*) and C 2p*_z_* orbitals, respectively. Under excitation, the electrons on N (2p*_x_*, 2p*_y_*) transfer to C 2p*_z_* orbitals should be difficult due to the different orbital directions. Moreover, in pure g‐C_3_N_4_, the excited electrons (from N atoms to C atoms) need to re‐transfer from C atoms to the N atoms (active sites) for catalytic reaction, which should be an important reason to explain the high recombination rate of photogenerated charge carriers in plane of C–N heterocycles. For B‐doped g‐C_3_N_4_, Figure 2e–g showed that the top of VB is mainly contributed by N (2p*_x_*, 2p*_y_*), while the bottom of CB is composed by C 2p*_z_* and B (2p*_x_*, 2p*_y_*) orbitals. The new electron pathway from N (2p*_x_*, 2p*_y_*) to B (2p*_x_*, 2p*_y_*) in the same plane is much easier than N (2p*_x_*, 2p*_y_*) to C 2p*_z_* (Figure 2h). This result can explain that the B‐doped g‐C_3_N_4_ changes the charge excitation and localization.

**Figure 1:**
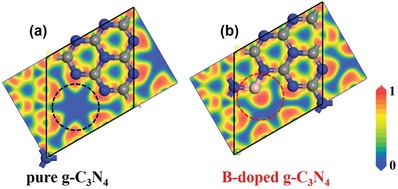
Electronic localization function of a) pure g‐C_3_N_4_ and b) B‐doped g‐C_3_N_4_ on the parallel plane. (The red areas represent high probability of electrons, while the blue areas represent low probability. The gray, blue, and pink spheres represent C, N and B atoms, respectively).

**Figure 2:**
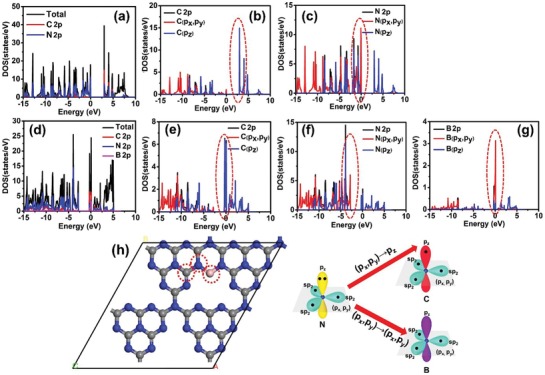
The calculated density of states (DOS) of a–c) pure g‐C_3_N_4_ and d–g) B‐doped g‐C_3_N_4_. h) Schematic diagram of electrons excited from N (2p*_x_*, 2p*_y_*) to C 2p*_z_* or B (2p*_x_*, 2p*_y_*). (The gray, blue, and pink spheres represent C, N, and B atoms, respectively).

Inspired by the theoretical prediction, B‐doped g‐C_3_N_4_ was prepared by a one‐step calcination of a mixture of boric acid and urea. **Figure** **3**a exhibits the typical transmission electron microscope (TEM) image of 1%B/g‐C_3_N_4_. Obviously, ultrathin lamellar graphene‐like structure can be observed,^29^, ^30^ which is similar to the morphology of pure g‐C_3_N_4_ (Figure S6, Supporting Information).

**Figure 3:**
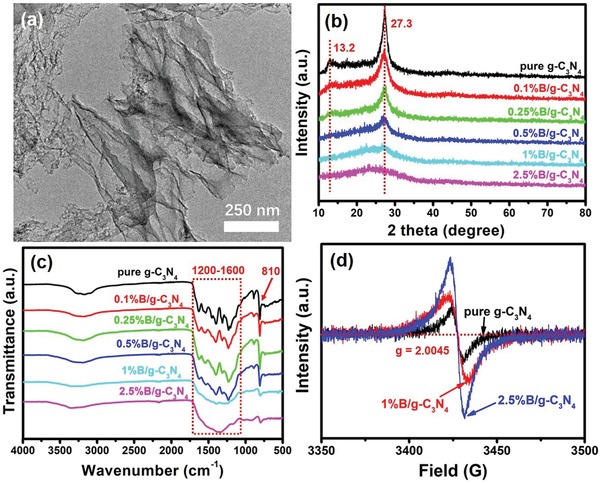
a) TEM images of 1%B/g‐C_3_N_4_. b) XRD and c) FTIR spectra of the as‐prepared samples. d) Room‐temperature ESR spectra of pure g‐C_3_N_4_, 1%B/g‐C_3_N_4_, and 2.5%B/g‐C_3_N_4_.

Energy‐dispersive X‐ray (EDX) elemental mapping images of 1%B/g‐C_3_N_4_ (Figure S7, Supporting Information) prove that B atoms are uniformly distributed. The atomic force microscope (AFM) image (Figure S8, Supporting Information) further proves the ultrathin structure with ≈1.2 nm thickness in 1%B/g‐C_3_N_4_. Figure 3b shows the XRD spectra of the samples. Clear characteristic peaks located at 13.2° and 27.3° can be observed, which can be assigned to the typical characteristic peaks of g‐C_3_N_4_.^2^, ^31^, ^32^ With increasing of the B‐doping content, these two characteristic peaks were getting weaker, indicating B‐doping changes the periodic arrangement of atoms in g‐C_3_N_4_. For 2.5%B/g‐C_3_N_4_ sample, only a small broad peak at 20°–30° can be observed, which is attributed to amorphous matter derived from chemical reaction of boric acid and urea.^14^, ^33^ The Fourier transform infrared (FTIR) spectra of the samples are shown in Figure 3c. Characteristic peaks of g‐C_3_N_4_ located at 1200–1600 and 810 cm^−1^ can be attributed to skeletal vibrations of aromatic C–N heterocycles and breathing vibration of triazine units, respectively.^34^ The doping of B influences the vibration modes of C–N structure in g‐C_3_N_4_. With the increasing of B‐doping, all the characteristic peaks of g‐C_3_N_4_ become weaker than pure g‐C_3_N_4_. Obviously, 2.5%B/g‐C_3_N_4_ exhibit different shapes of FTIR spectrum compared with other samples. A new peak located at 1350 cm^−1^ appears, and the peak at 1230 cm^−1^ disappears (partial enlarged spectra shown in Figure S9 in the Supporting Information). The former belongs to the in‐plane B–N stretching vibration of BN,^35^ while the latter belongs to the typical stretching vibration modes of C=N or C—N in the heterocycles of g‐C_3_N_4_.^36^ This result means that too much B‐doping caused the decay of g‐C_3_N_4_ and resulted amorphous BN in 2.5%B/g‐C_3_N_4_. Figure S10 (Supporting Information) exhibits the TEM images of 2.5%B/g‐C_3_N_4_. Clearly, typical nanosheet structures can be observed without visible lattice fringe, which further confirm the presence of amorphous BN in the 2.5%B/g‐C_3_N_4_ sample. For further investigating the structure changes brought by B‐doping, electron paramagnetic resonance (EPR) spectra of pure g‐C_3_N_4_, 1%B/g‐C_3_N_4_, and 2.5%B/g‐C_3_N_4_ were performed (Figure 3d). Primary Lorentzian lines with *g* value of 2.00 can be observed in three samples. These signals come from the unpaired electrons on the sp^2^‐nitrogen atoms of the π‐conjugated C–N aromatic rings.^37^, ^38^ The intensities of signal peak become stronger after B‐doping, indicating higher concentration of unpaired electrons in B‐doped g‐C_3_N_4_.

**Figure** **4**a,b compares the C 1s and N 1s XPS spectra of pure g‐C_3_N_4_ and 1%B/g‐C_3_N_4_. The XPS survey spectra and B 1s spectra (Figure S11, Supporting Information) fully prove the existence of B in 1%B/g‐C_3_N_4_. Table S1 (Supporting Information) exhibits that the actual content of B in 1%B/g‐C_3_N_4_ was measured to be 5.06 at%. In Figure 4a, the C 1s spectrum of pure g‐C_3_N_4_ can be fitted into three peaks. The peak at 284.8 eV is assigned to potential surface impurities of sp^2^ carbon. The peak at 288.1 eV is belong to the carbon in N=C—N_2_. A very small peak located at 286.2 can be attributed to the carbon in terminal cyano (C≡N).^34^ For 1%B/g‐C_3_N_4_, just two peaks (284.8 and 287.9 eV) can be observed. The disappearance of peak located at 286.2 eV can be attributed to the fact that B is more easily to coordinate with N and thus reduce the terminal cyano group. Slight negative shift (from 288.1 to 287.9 eV) of C in N=C‐N_2_ is because the electronegativity order of the elements is B (2.0) < C (2.5) < N (3.0). In Figure 4b, pure g‐C_3_N_4_ shows three peaks located at 398.7, 400.0, and 401.1 eV, which can be assigned to N in two‐coordinated N (C=N—C), three‐coordinated N (N—(C)_3_), and surface amino (N—H species), respectively.^14^, ^30^ For 1%B/g‐C_3_N_4_, no apparent signal of surface amino can be detected, but only two peaks of 398.4 and 399.7 eV attributed to two‐coordinated and three‐coordinated N are observed.^34^ More interestingly, the ratio of two‐coordinated N to three‐coordinated N decreased obviously, indicating that B was doped into the large cavity and bonded with the two‐coordinated N.

**Figure 4:**
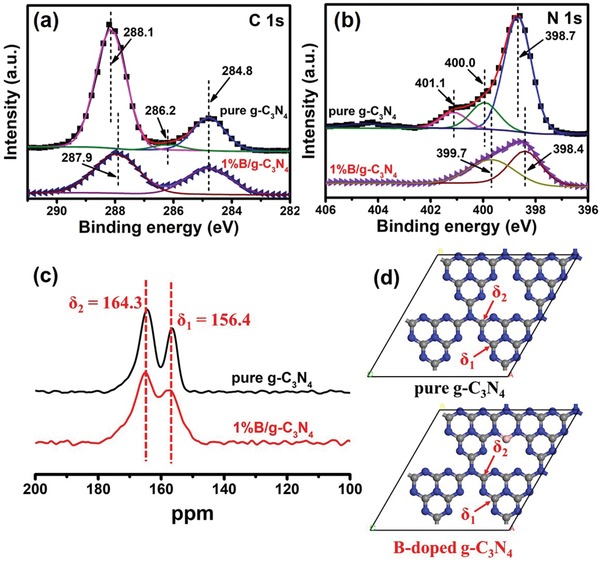
High‐resolution XPS spectra of a) C 1s and b) N 1s in pure g‐C_3_N_4_ and 1%B/g‐C_3_N_4_. c) Solid‐state ^13^C magic angle spinning NMR of pure g‐C_3_N_4_ and 1%B/g‐C_3_N_4_. d) Possible molecular configuration of pure g‐C_3_N_4_ and B‐doped g‐C_3_N_4_.

To confirm the actual doping site of B atoms, solid ^13^C NMR analyses were conducted. Figure 4c shows that both pure g‐C_3_N_4_ and 1%B/g‐C_3_N_4_ exhibit two obvious peaks located at 156.4 and 164.3 ppm, which are ascribed to the chemical shifts of C at the different position of C–N aromatic rings (Figure 4d).^39^, ^40^, ^41^, ^42^ No new peak appears in 1%B/g‐C_3_N_4_, indicating B did not directly coordinate with C. Moreover, as shown in Figure S12 (Supporting Information), obvious peak located at 7.66 ppm can be ascribed to the ‐BN_2_. This result directly proves that the B atoms connected with N atoms, not C atoms. Combined with the XPS results, the possible molecular configuration of 1%B/g‐C_3_N_4_ is shown in Figure 4d.

The light absorption properties of samples were studied by UV–visible spectroscopy (Figure S13a, Supporting Information). Pure g‐C_3_N_4_ shows a clear absorption band edge at 460 nm, indicating a bandgap of 2.7 eV.^43^ After B‐doping, obvious tailing peaks can be observed in the range of 450–550 nm. 1%B/g‐C_3_N_4_ shows small redshift of absorption band edge, indicating a slight narrowing of the bandgap. For 2.5%B/g‐C_3_N_4_, slight blueshift can be observed. The amorphous BN, a wide‐bandgap semiconductor, can explain the slight blueshift of absorption band edge.^44^ Figure S13b (Supporting Information) compares the N_2_ adsorption‐desorption isotherm of pure g‐C_3_N_4_, 1%B/g‐C_3_N_4_, and 2.5%B/g‐C_3_N_4_. The detailed data of specific surface area, average pore size, and pore volume are shown in Table S2 (Supporting Information). Compared with pure g‐C_3_N_4_ (41 m^2^ g^−1^), both 1%B/g‐C_3_N_4_ (62 m^2^ g^−1^), and 2.5%B/g‐C_3_N_4_ (80 m^2^ g^−1^) exhibit higher specific surface area. The high specific surface area of 2.5%B/g‐C_3_N_4_ is due to the contribution of amorphous BN. Slightly increase in light absorption property and specific surface area indicate that other more critical factors affect the photocatalytic activity.

We tend to investigate the charge transfer dynamics using photoluminescence (PL) spectra. As the B doping content increases, the PL emission intensity of B‐doped g‐C_3_N_4_ at 460 nm greatly decreases (**Figure** **5**a), indicating B‐doping can bring better charge transfer.^45^ In order to further study the properties of photogenerated charges, time‐resolved photoluminescence (TRPL) spectra are performed (Figure 5b). Compared with pure g‐C_3_N_4_, both 0.5%B/g‐C_3_N_4_ and 1%B/g‐C_3_N_4_ exhibit obvious longer fluorescence lifetime, confirming efficient charge transfer and localization for photocatalytic reactions.^46^, ^47^ While, the fluorescence lifetime of 2.5%B/g‐C_3_N_4_ (Figure 5b) is much shorter than pure g‐C_3_N_4_, indicating that appropriate B amount is extremely important for optimizing the charge transfer and localization.

**Figure 5:**
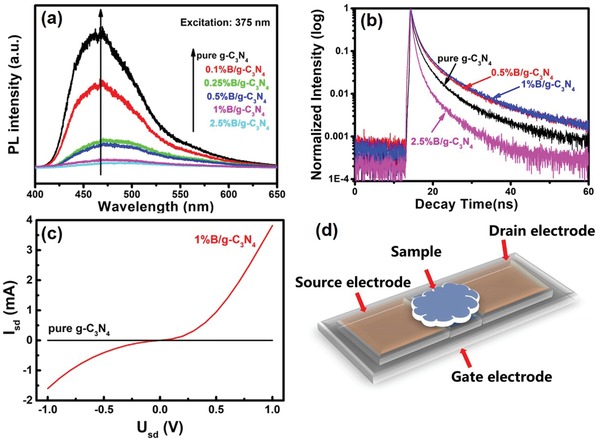
a) PL and b) TRPL spectra of pure g‐C_3_N_4_, 1%B/g‐C_3_N_4_ and 2.5%B/g‐C_3_N_4_. c) Output characteristic curves of pure g‐C_3_N_4_ and 1%B/g‐C_3_N_4_. d) Schematic diagram of output characteristic curves tests.

The improvement of the charge transfer dynamics is further confirmed by the measurement on a physics property measurement system (PPMS) (Figure 5c). As shown in Figure 5d, the samples were coated on the three‐electrode transistor device. Output characteristic curves were obtained by measuring the current signal between the source (s) electrode and drain (d) electrode with applied voltage. Compared with pure g‐C_3_N_4_, 1%B/g‐C_3_N_4_ exhibits great enhancement of electric conductivity. Better electric conductivity confirms better charge transfer.

Next, photocatalytic reduction of CO_2_ is carried out to verify whether B improve the reaction dynamics. **Figure** **6**a exhibits the photocatalytic CH_4_ yield of CO_2_ reduction with the as‐prepared samples. Figure S14 (Supporting Information) shows CO has higher priority than CH_4_ in pure g‐C_3_N_4_. As we known, the reaction from CO_2_ to CH_4_ is an 8‐electron reduction reaction. Low surface density of photogenerated electrons kinetically limits the reaction rate of CO_2_ to CH_4_. The CH_4_ yield of B‐doped g‐C_3_N_4_ is greatly improved on B‐doped g‐C_3_N_4_. The optimal sample of 1%B/g‐C_3_N_4_ exhibits about 32 times higher CH_4_ yield than pure g‐C_3_N_4_, indicating the improvement of the reaction dynamics.

**Figure 6:**
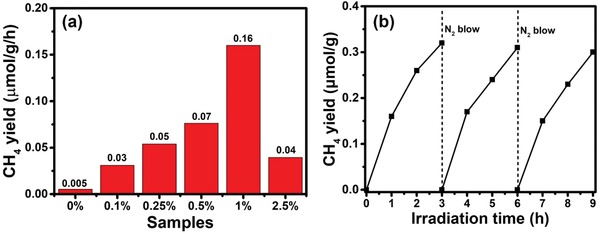
a) Photocatalytic CH_4_ yield of CO_2_ reduction with the as‐prepared samples (*x*% represents *x*%B/g‐C_3_N_4_, 0% represents pure g‐C_3_N_4_). b) Time courses of photocatalytic activity for CH_4_ production over 1%B/g‐C_3_N_4_.

In order to further study the reaction dynamics, the CO temperature programmed desorption (TPD) measurements are conducted and CO adsorption energies are deduced (Figure S15 and Table S3, Supporting Information). As shown in Figure S15 (Supporting Information), 1%B/g‐C_3_N_4_ exhibited significantly higher CO desorption temperature than that of pure g‐C_3_N_4_, indicating the B‐doped g‐C_3_N_4_ has high electron density to adsorb CO. The adsorbed CO molecules can be further reduced to generate CH_4_, which explains the higher CH_4_ production rate of B‐doped g‐C_3_N_4_. Table S3 (Supporting Information) shows that the CO adsorption energy of the B‐doped g‐C_3_N_4_ is more negative than the pure g‐C_3_N_4_, which is consistent with the results of CO refractory desorption. We have also studied the CO_2_ reduction mechanism on the pure g‐C_3_N_4_ (Figure S16, Supporting Information). The adsorption free energy of CO_2_ is 0.11 eV, indicating that the interaction between CO_2_ and g‐C_3_N_4_ is physisorption. The determining‐rate step is the formation of COOH*, and the free energy barrier is 1.27 eV (1.38 − 0.11 = 1.27). These results suggest the CO_2_ reduction reaction is difficult to perform on the surface of g‐C_3_N_4_. On the contrary, the CO_2_ adsorption on the B site is more stable than other site, and the adsorption free energy is −2.2 eV. We further calculated the reduction Gibbs free energies along the optimal path for CO_2_ reduction to CH_4_. These results show that the determining‐rate step is formation CH_4_*, and the energy barrier is only 0.52 eV (−3.40 − (−3.92) = 0.52). B‐doped g‐C_3_N_4_ can facilitate the activation of CO_2_ and enhance the selectivity of CO_2_ reduction to CH_4_. Moreover, no obvious performance degradation over the three cycles (Figure 6b), proving the high stability of 1%B/g‐C_3_N_4_. Isotope tracer experiments were used to identify the carbon source of products and tested by GC‐MS (Figure S17, Supporting Information). ^12^CO_2_ gas was used as a reference. Clearly, only ^12^CH_4_ and ^12^CO can be detected when ^12^CO_2_ as reactant. Intense signal corresponding to ^13^CH_4_ (*m*/*z* = 17) and ^13^CO (*m*/*z* = 29) with ^13^CO_2_ as the carbon source can strongly supports that the detected product (CH_4_ and CO) are from photoreduction of CO_2_ reactant, not other surface contaminant carbon species.

In summary, we first analyzed the reasons for the low charge transfer and reaction dynamics in pure g‐C_3_N_4_ and predicted that B‐doping can improve the charge transfer and localization by DFT theoretical calculations. By calcination mixture of boric acid and urea, B atom was doped in the large cavity between adjacent tri‐s‐triazine units and coordinated with two‐coordinated N in g‐C_3_N_4_. Theoretical calculations prove that new electron excitation from N (2p*_x_*, 2p*_y_*) to B (2p*_x_*, 2p*_y_*) with the same orbital direction in B‐doped g‐C_3_N_4_ is much easier than N (2p*_x_*, 2p*_y_*) to C 2p*_z_* in pure g‐C_3_N_4_. Moreover, B atoms doping changes the adsorption of CO (intermediate), and can act as active sites for CH_4_ production. As a result, the optimal sample of 1%B/g‐C_3_N_4_ exhibited about 32 times higher CH_4_ (an 8‐electron reduction product) yield than pure g‐C_3_N_4_. This work provides a new insight to tune the intrinsic charge localization and improve interior charge transfer and reaction dynamics properties of g‐C_3_N_4_ for better photocatalytic performance.

## Conflict of Interest

The authors declare no conflict of interest.

## Supporting information

Supplementary
