# Supplementary material for: Graphitic Carbon Nitride with Dopant Induced Charge Localization for Enhanced Photoreduction of CO2 to CH4
Source: Adv Sci (Weinh). 2019 Jul 26;6(18):1900796. doi: 10.1002/advs.201900796 (PMC6755511; doi:10.1002/advs.201900796)
Supplement: Supplementary file 1 — Supplementary [file ADVS-6-1900796-s001.pdf]

Copyright WILEY-VCH Verlag GmbH & Co. KGaA, 69469 Weinheim, Germany, 2019.

ADVANCED SCIENCE
Open Access

Supporting Information

for *Adv. Sci.,* DOI: 10.1002/advs.201900796

Graphitic Carbon Nitride with Dopant Induced Charge Localization for Enhanced Photoreduction of CO_2 to CH_4

Junwei Fu, Kang Liu, Kexin Jiang, Huangjingwei Li, Pengda An, Wenzhang Li, Ning Zhang, Hongmei Li, Xiaowen Xu, Haiqing Zhou, Dongsheng Tang, Xiaoming Wang, Xiaoqing Qiu,* and Min Liu**

Copyright WILEY-VCH Verlag GmbH & Co. KGaA, 69469 Weinheim, Germany, 2019.

Supporting Information

Graphitic Carbon Nitride with Dopant Induced Charge Localization for Enhanced Photoreduction CO_2 to CH_4

Junwei Fu+, Kang Liu+, Kexin Jiang, Huangjingwei Li, Pengda An, Wenzhang Li, Ning Zhang, Haiqing Zhou, Dongsheng Tang, Xiaoming Wang, Xiaoqing Qiu,* and Min Liu**

Table of Contents

1. Experimental Procedures 2
1.1. Sample preparation 2
1.2. Characterizations 2
1.3. Computational details 2
1.4. Photocatalytic reduction of CO_2 3
2. Results and Discussion 4
Figure S1. The density of states of edge atoms for (a, b) pure g-C_3N_4 and (d, e) B-doped g-C_3N_4. (c) and (f) is the structure diagram of pure g-C_3N_4 and B-doped g-C_3N_4, respectively. 4
Figure S2. The partial charge density distribution of initial state (left) and final state (right) for (a,b) pure g-C_3N_4 and (c,d) B-doped g-C_3N_4. 6
Figure S3. The project density of states of N, C, S for the S-doped g-C_3N_4. 7
Figure S4. The project density of states of N, C, P for the P-doped g-C_3N_4. 8
Figure S5. Electronic localization functions of P-doped g-C_3N_4 (a) and S-doped g-C_3N_4 (b) on the parallel plane. 9
Figure S6. TEM image of the pure g-C_3N_4. 10
Figure S7. Energy-dispersive X-ray (EDX) elemental mapping images of 1%B/g-C_3N_4. 11
Figure S8. AFM image of 1%B/g-C_3N_4. 12
Figure S9. Partial enlarged FTIR spectra (1000–1750 cm^{-1}) of samples. 13
Figure S10. (a,b) TEM images of 2.5%B/g-C_3N_4 with different magnification. 14
Figure S11. The XPS survey spectra (a) and B 1s spectra (b) of the pure g-C_3N_4 and 1%B/g-C_3N_4. 15
Figure S12. Solid-state ^{11}B magic angle spinning NMR spectrum of 1%B/g-C_3N_4. 16
Figure S13. UV-vis DRS (a) and N_2 adsorption-desorption isotherm (b) of pure g-C_3N_4, 1%B/g-C_3N_4 and 2.5%B/g-C_3N_4. 17
Figure S14. Photocatalytic CO yield of CO_2 reduction with the as-prepared samples (x% represents x%B/g-C_3N_4, 0% represents pure g-C_3N_4). 18
Figure S15. Temperature program desorption (TPD) curves of CO on the surface of pure g-C_3N_4, and 1%B/g-C_3N_4. 19
Figure S16. The free energy diagram corresponding to the optimal path for CO_2 reduction reaction on the (a) pure g-C_3N_4 and (b) B-doped g-C_3N_4. 20
Figure S17. Mass spectra of the product obtained by the photocatalytic reduction of $^{12}CO_2$ and $^{13}CO_2$ over 1%B/g-C_3N_4. 21
Table S1. The atomic percentage of C, N, B and O in the sample 1%B/g-C_3N_4 and pure g-C_3N_4 measured by XPS data. 22
Table S2. The specific surface area, average pore size and pore volume of pure g-C_3N_4, 1%B/g-C_3N_4 and 2.5%B/g-C_3N_4. 23
Table S3. The calculated adsorption energy of CO on pure g-C_3N_4 and B-doped g-C_3N_4. 24
References 25

1. Experimental Procedures

1.1. Sample preparation

B-doped g-C_3N_4 samples were synthesized by a one-step calcination of a mixture of boric acid and urea. In detail, 10 g of urea and a certain amount of boric acid were mixed evenly and packed in a covered crucible. The crucible was heated in a muffle furnace at 550 °C for 2 h with a 5 °C/min heating rate. The obtained yellow product was ground for backup use. The samples with different B doping contents were named x%B/g-C_3N_4, x% are the relative mass ratio of boric acid and urea.

1.2. Characterizations

Transmission electron microscope (TEM) images were obtained on a FEI Tecnai G2 F20 S-Twin probe corrector microscope. Atomic force microscope (AFM) image was taken with SPM-9700HT, (Shimadzu, Japan). X-ray diffraction (XRD) patterns were collected using a D8 advance X-ray diffractometer (Rigaku, Japan). Fourier transform infrared (FTIR) spectra were recorded on a Nicolet 6700 (Thermo, USA) spectrometer. Electron spin resonance (ESR) signals were recorded at room temperature on a Bruker A300 spectrometer. X-ray photoelectron spectroscopy (XPS) were performed on Thermo Fisher Scientific-Escalab 250Xi. All the binding energies were calibrated by the C 1s peak at 284.8 eV. Solid state C^{13} nuclear magnetic resonance (NMR) was measured on an Agilent 600M spectrometer. UV–visible diffuse reflection spectra were obtained on a UV–visible spectrophotometer (UV-2600, Shimadzu, Japan) with $BaSO_4$ as the reflectance sample. Nitrogen adsorption desorption isotherms were obtained on Micromeritics ASAP 2020 nitrogen adsorption apparatus. Photoluminescence (PL) measurement was performed on Horiba LabRAM HREVO with excitation of 365 nm. Time resolved fluorescence spectra were obtained on single photon counting (TCSPC) system (Picoquant "Timeharp 300") at wavelength of 450 nm. Output characteristic curves were measured on Keithley 4200 Semiconductor Characterization System. CO_2 adsorption isotherms were recorded on Micromeritics ASAP 3020 adsorption apparatus. CO temperature program desorption (TPD) curves were measured on Micromeritics AutoChem 2920.

1.3. Computational details

All DFT calculations were carried out using the Vienna ab initio simulation package (VASP).[1] The Perdew−Burke−Ernzerh of exchange-correlation functional was used for the generalized gradient approximation.[2,3] All geometry optimizations were carried out with a cutoff energy of 450 eV and a Monkhorst-Pack k-point mesh of 5 × 5 × 1. The iterative process considered was convergences, when the force on the atom was less than 0.01 eV $Å^{-1}$ and the energy change was less than 10^{-5} eV per atom. The Heyd-Scuseria-Ernzerhof (HSE06) method was employed to investigate the electronic properties, and obtain an accurate description of density of states (DOS).[4-6]

The PBE+D2 method with the Grimme van der Waals correction was considered in calculation.[7] The free energy was defined as

$$G = E + E_{ZPE} - T \cdot S$$

Where E is the total energy from DFT calulation, E_{ZPE} is the zero-point energy, T is the temperature (298.15 K) and S is the enropy. Zero-point energies and entropies of the intermediates obtained from the vibrational frequencies.[8]

The adsorption energies (E_{ads}) of CO on the g-C_3N_4 and B-doped g-C_3N_4 modes are respectively obtained with the following equations.

$$E_{\mathrm{ads}}(\mathrm{CO}) = E(CO/Sub) - E(Sub) - E(CO)$$

Where E(CO/Sub) and E(Sub) are the total energies of the different systems with and without CO molecule, and E(CO) is the energy of an isolated CO molecule. From this definition, a more negative value of E_{ads} indicates that the adsorption is thermodynamically more stable and favorable.

1.4. Photocatalytic reduction of CO_2

Photocatalytic reduction of CO_2 was carried out by gas-solid surface reactions. CO_2 and H_2O vapor were come from the chemical reaction of H_2SO_4 solution and $NaHCO_3$ powder. Specific operation process can refer to the previous works.[9-11] Gas products were tested by gas chromatography (GC-2014c, Shimadzu, Japan) with a thermal conductivity detector (TCD) and a flame ion detector (FID). The gas composition and concentration were calibrated by the mixture standard gas. A 300 W Xe arc lamp (Microsolar 300, Perfectlight Science Co. LtD, China) was used as UV/visible light source.

2. Results and Discussion

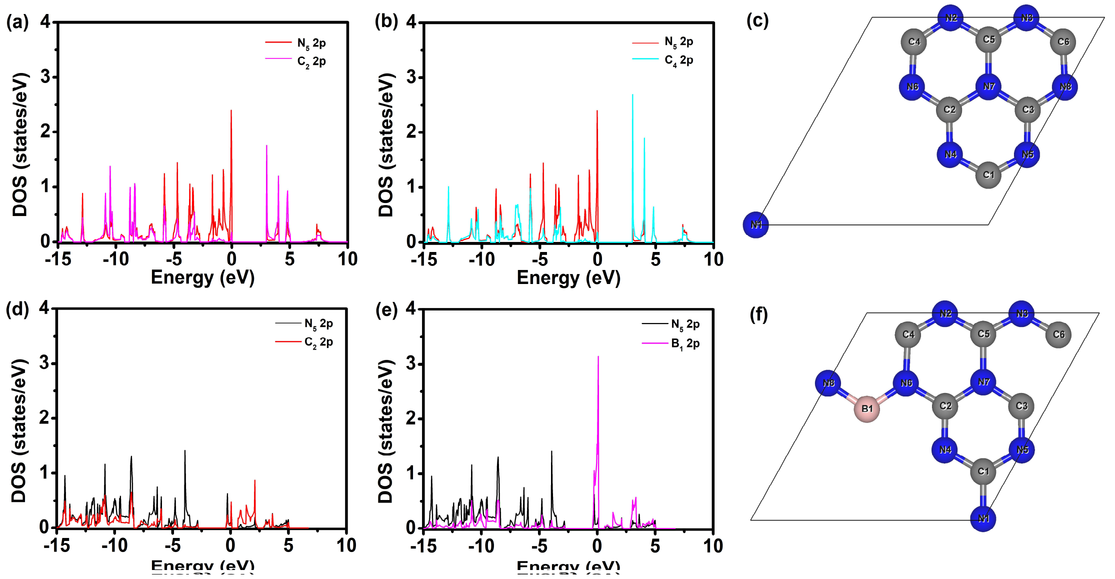

Figure S1. The density of states of edge atoms for (a, b) pure g-C_3N_4 and (d, e) B-doped g-C_3N_4. (c) and (f) is the structure diagram of pure g-C_3N_4 and B-doped g-C_3N_4, respectively.

The Fermi's golden rule is the transition probability (λ_{if}) from one initial state $|i\rangle$ to a set final state $|f\rangle$.

$$\lambda_{if} = \frac{2\pi}{\hbar}\left|M_{if}\right|^2 \rho_f$$

Where $\left|M_{if}\right|$ is the matrix element of the perturbation H' between the final and initial states and ρ_f is the density of states at the Fermi energy of the final states.

We don't know how to calculate the exact value of the matrix element ($\left|M_{if}\right|$). Based on the above formula, we focus on the density of states (DOS) of the final state, which can approximately indicate the transition probability (λ_{if}). We calculated the electronic structure of initial state and final state. For the DOS of g-C_3N_4 (Figure S1a and S1b), we further confirm the excitation of electrons from the two-coordinated N 2p orbitals (initial state) to C 2p orbitals (final state), for that the excited state (final state) is mainly contributed by the C 2p orbitals. For the DOS of B-doped g-C_3N_4 (Figure S1d and S1e), we can observe the electrons of highest occupied states come from the N 2p orbitals (initial state) and the excited states (final state) is composed of B 2p, N 2p and C 2p orbitals. We found B 2p orbitals

provided the main contribution on the excited state. Therefore, we propose that electrons are easily excited from N 2p to B 2p.

Moreover, we also calculated the partial charge density distribution of the initial and final state (Figure S2). The electrons of initial state are mainly localized on two-coordinated N atoms and distributed in the x, y plane in both g-C_3N_4 and B-doped g-C_3N_4. The final state is mainly distributed in the z direction of C and N atoms for pure g-C_3N_4. Thus, the electrons excite from N ($2p_x$, $2p_y$) to C $2p_z$ orbitals in pure g-C_3N_4. Differently, we found the final state in B-doped g-C_3N_4 are almost localized on B atom along the x, y plane. These results show the electrons excite from N ($2p_x$, $2p_y$) to B ($2p_x$, $2p_y$) orbitals is superior to original excite pathway (from N ($2p_x$, $2p_y$) to C $2p_z$ orbitals) in B-doped g-C_3N_4. Therefore, the new electron pathway from N ($2p_x$, $2p_y$) to B ($2p_x$, $2p_y$) in the same plane is much easier than N ($2p_x$, $2p_y$) to C $2p_z$.

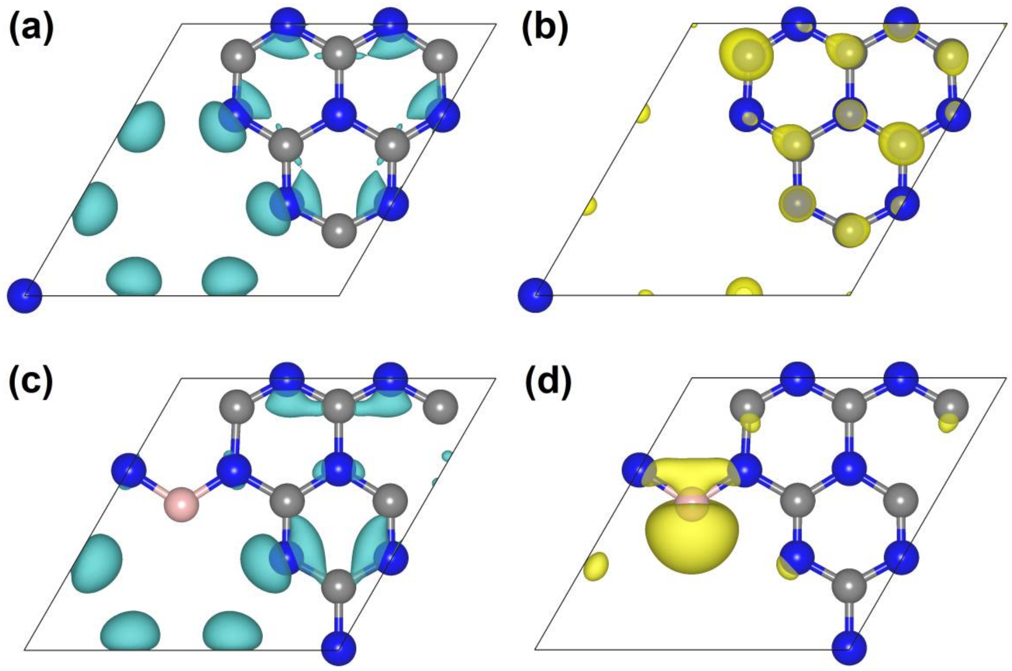

Figure S2. The partial charge density distribution of initial state (left) and final state (right) for (a,b) pure g-C_3N_4 and (c,d) B-doped g-C_3N_4.

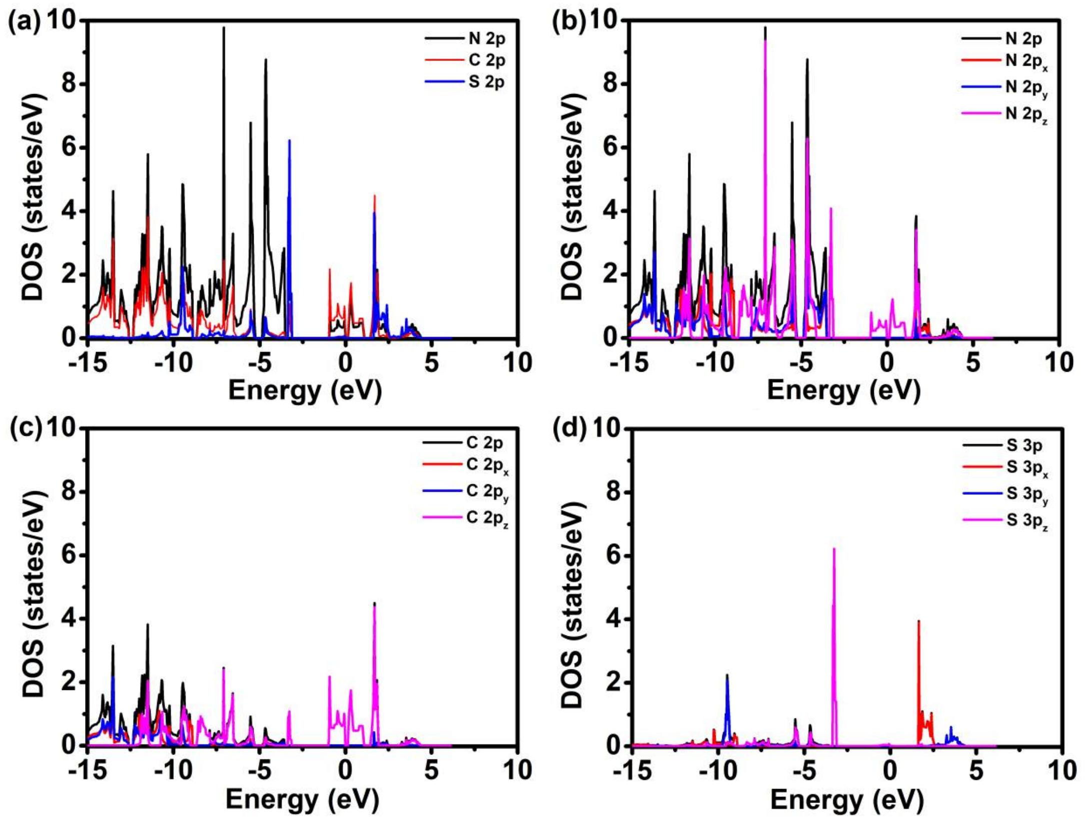

Figure S3. The project density of states of N, C, S for the S-doped g-C_3N_4.

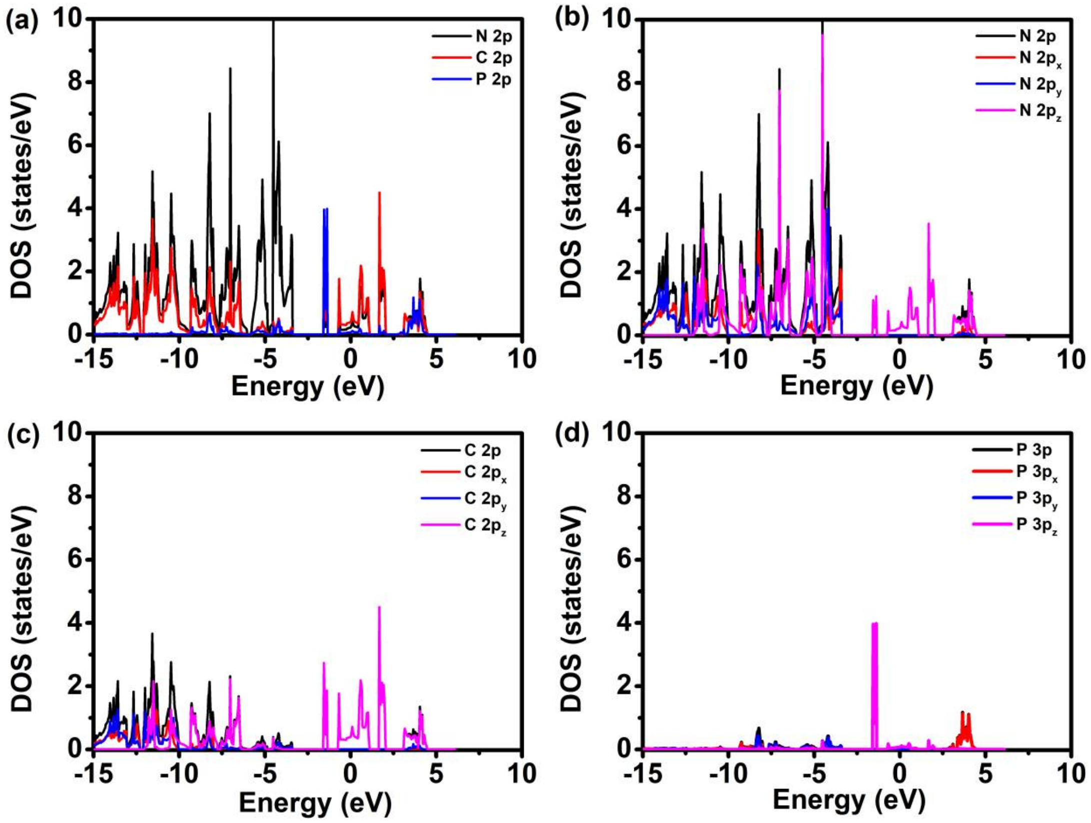

Figure S4. The project density of states of N, C, P for the P-doped g-C_3N_4.

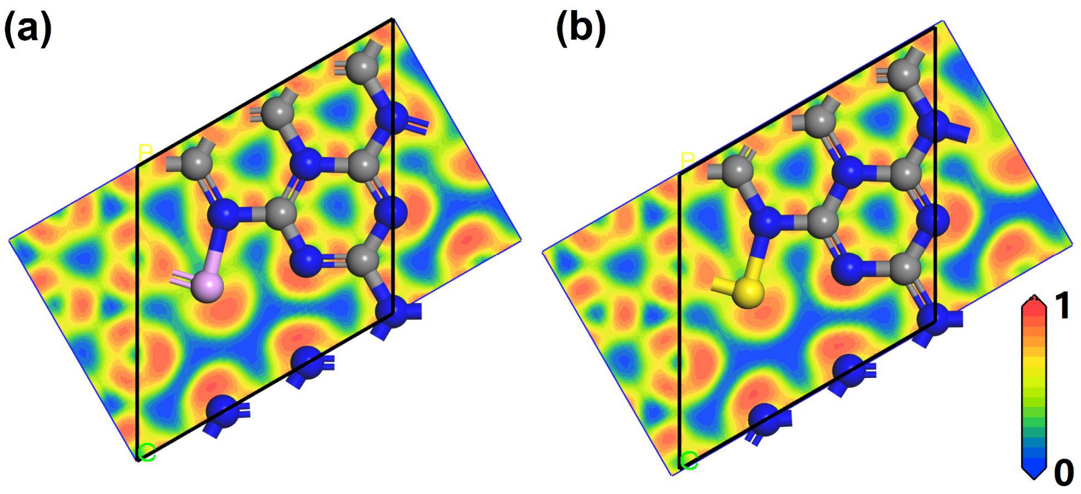

Figure S5. Electronic localization functions of P-doped g-C_3N_4 (a) and S-doped g-C_3N_4 (b) on the parallel plane.

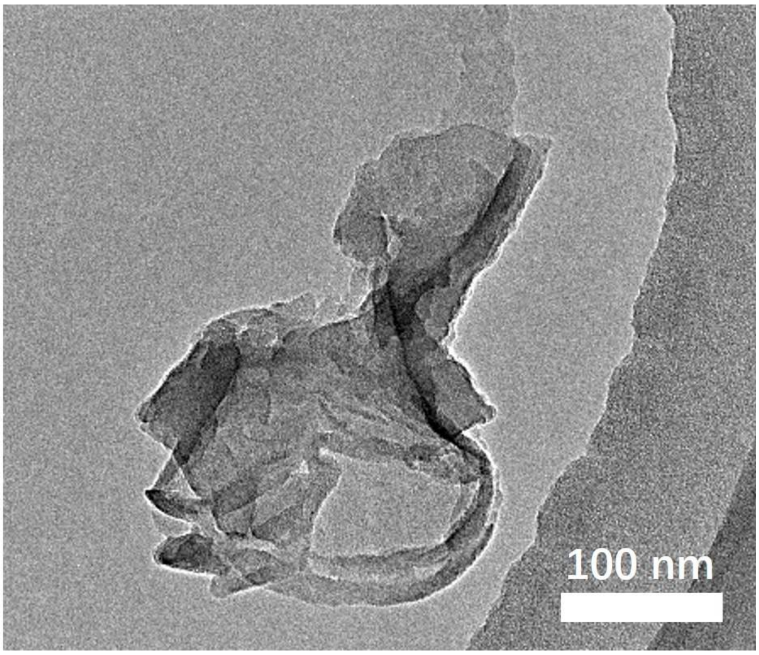

Figure S6. TEM image of the pure g-C_3N_4.

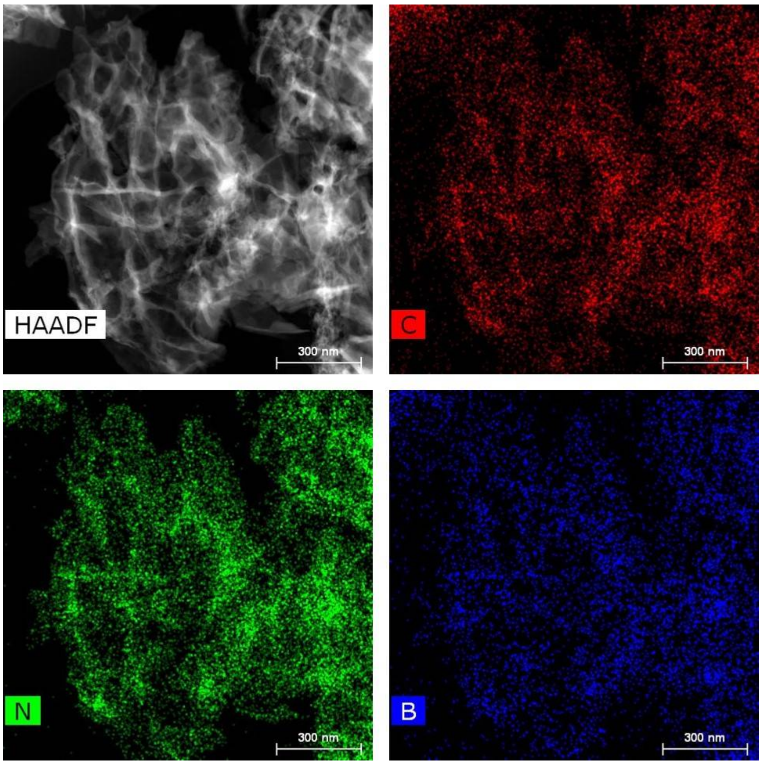

Figure S7. Energy-dispersive X-ray (EDX) elemental mapping images of 1%B/g-C_3N_4.

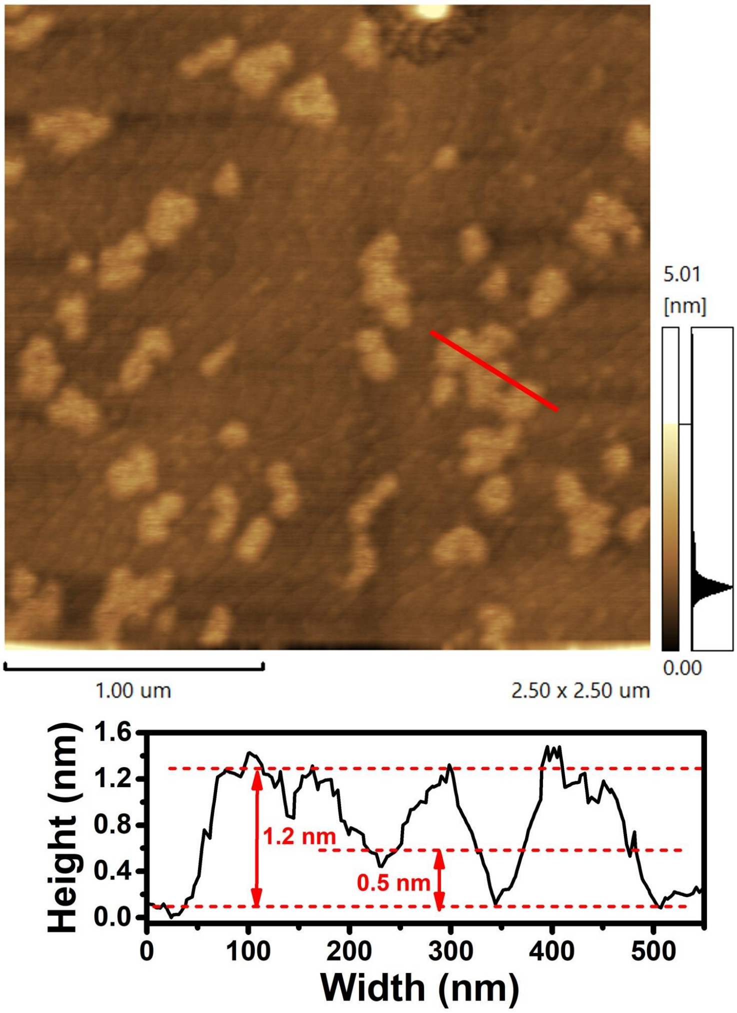

Figure S8. AFM image of 1%B/g-C_3N_4.

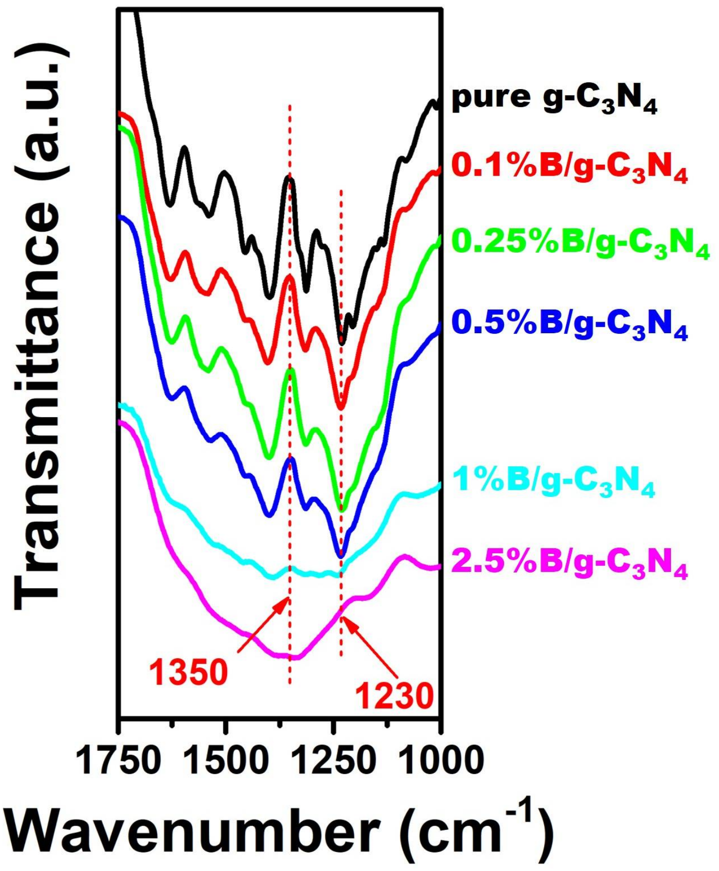

Figure S9. Partial enlarged FTIR spectra (1000–1750 cm^{-1}) of samples.

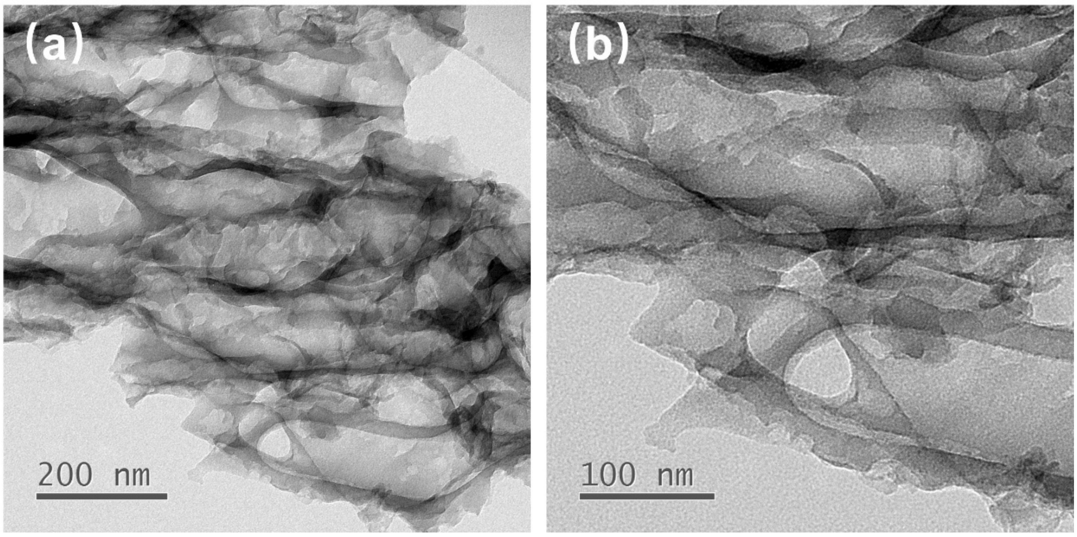

Figure S10. (a,b) TEM images of 2.5%B/g-C_3N_4 with different magnification.

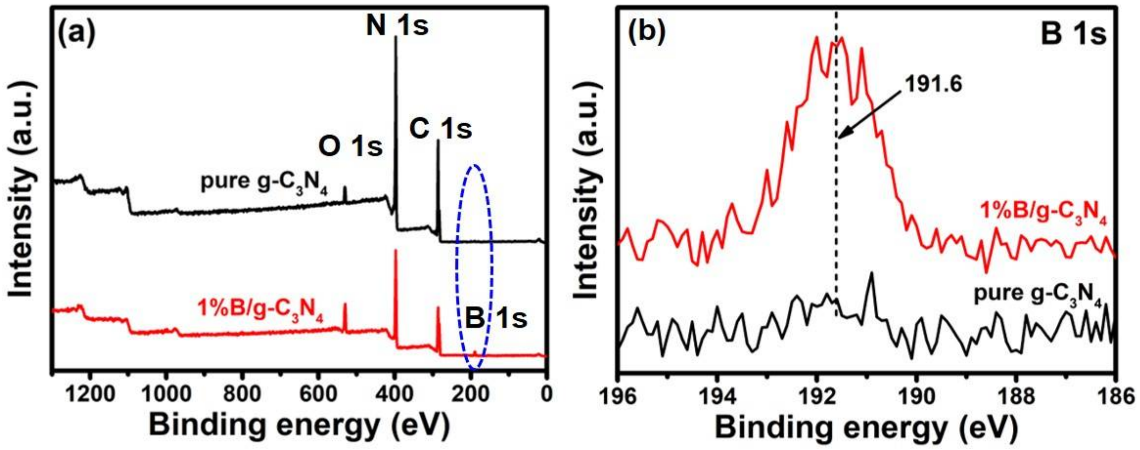

Figure S11. The XPS survey spectra (a) and B 1s spectra (b) of the pure g-C_3N_4 and 1%B/g-C_3N_4.

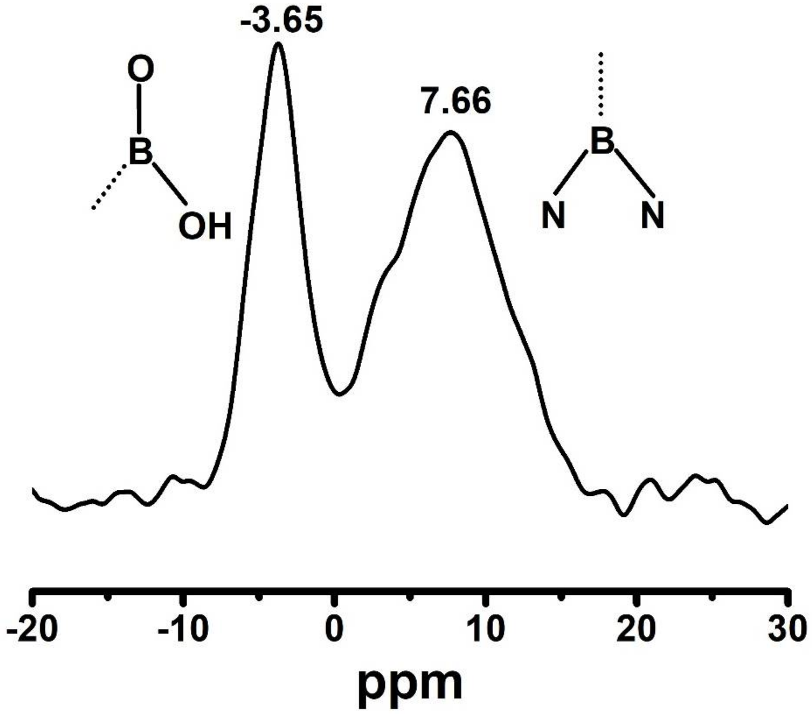

Figure S12. Solid-state ^{11}B magic angle spinning NMR spectrum of 1%B/g-C_3N_4.

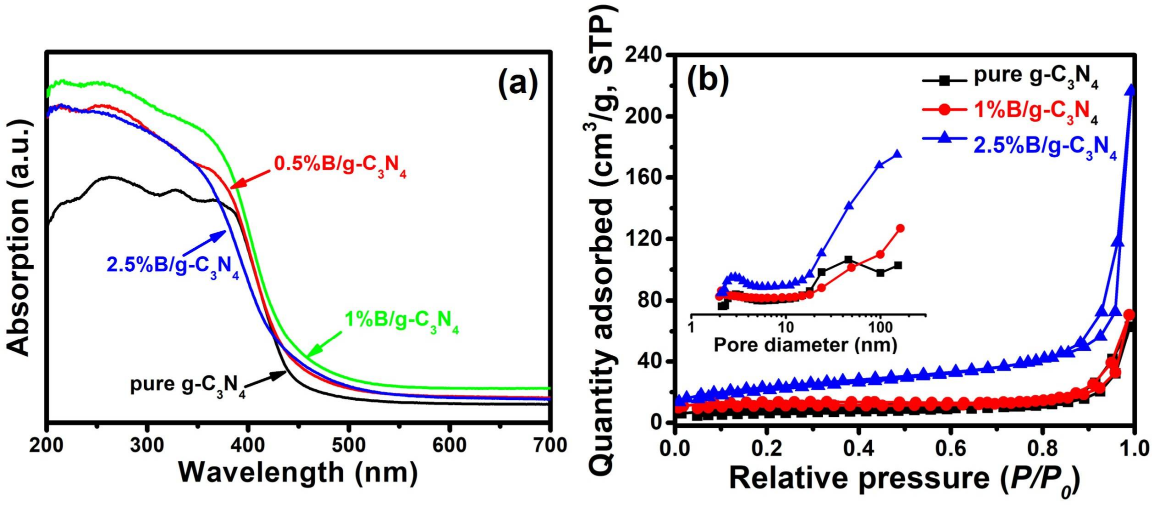

Figure S13. UV-vis DRS (a) and N_2 adsorption-desorption isotherm (b) of pure g-C_3N_4, 1%B/g-C_3N_4 and 2.5%B/g-C_3N_4.

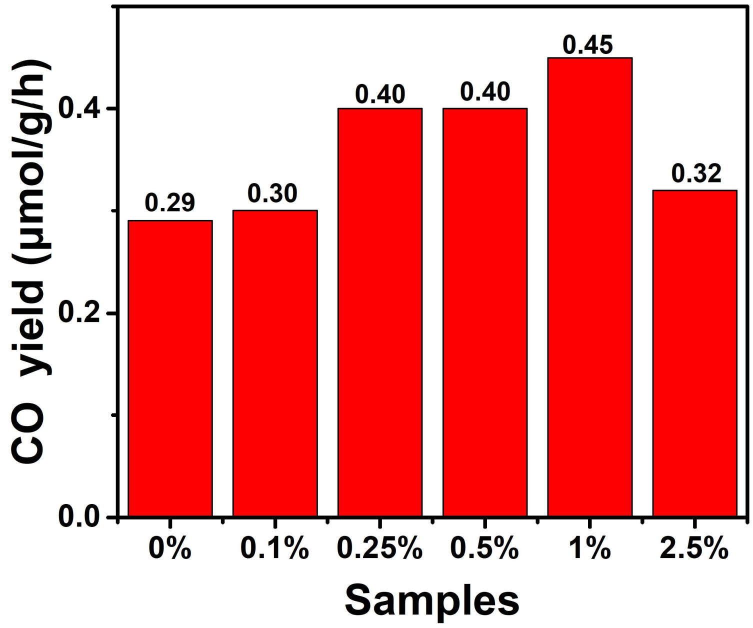

Figure S14. Photocatalytic CO yield of CO_2 reduction with the as-prepared samples (x% represents x%B/g-C_3N_4, 0% represents pure g-C_3N_4).

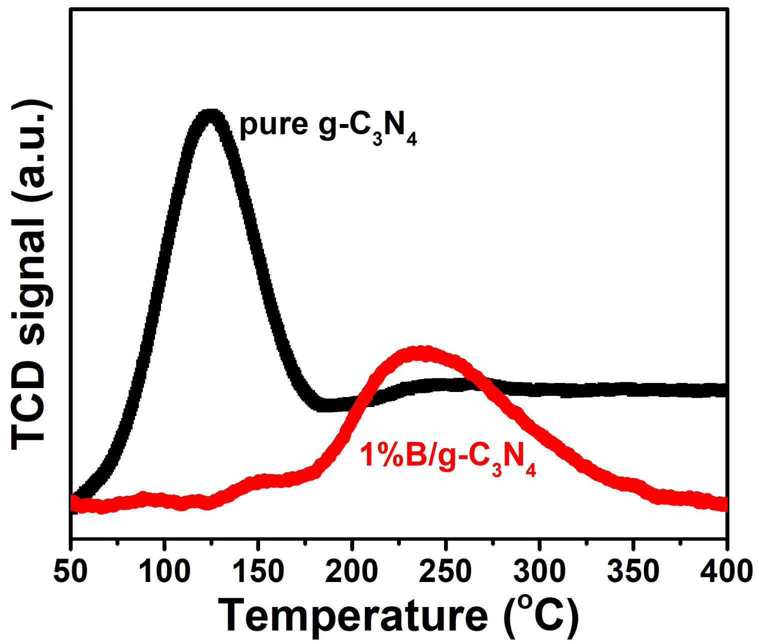

Figure S15. Temperature program desorption (TPD) curves of CO on the surface of pure g-C_3N_4, and 1%B/g-C_3N_4.

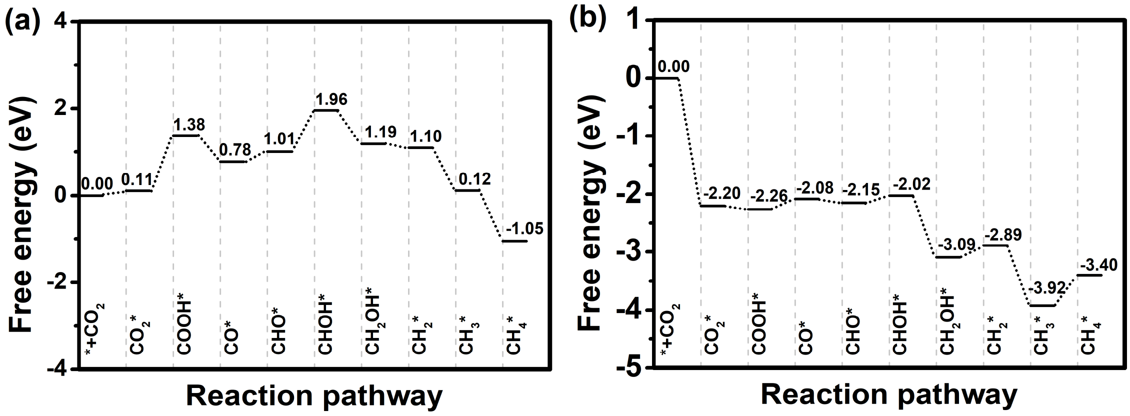

Figure S16. The free energy diagram corresponding to the optimal path for CO_2 reduction reaction on the (a) pure g-C_3N_4 and (b) B-doped g-C_3N_4.

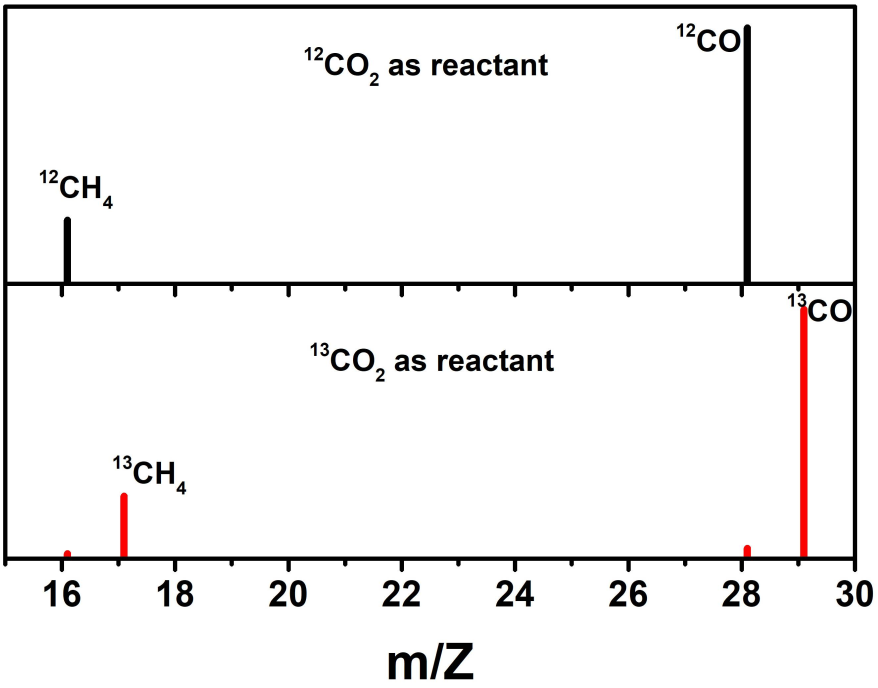

Figure S17. Mass spectra of the product obtained by the photocatalytic reduction of $^{12}CO_2$ and $^{13}CO_2$ over 1%B/g-C_3N_4.

Table S1. The atomic percentage of C, N, B and O in the sample 1%B/g-C_3N_4 and pure g-C_3N_4 measured by XPS data.

Sample	C (at.%)	N (at.%)	O (at.%)	B (at.%)
1%B/g-C_3N_4	44.38	44.30	6.26	5.06
pure g-C_3N_4	46.03	49.34	4.63	-

Table S2. The specific surface area, average pore size and pore volume of pure g-C_3N_4, 1%B/g-C_3N_4 and 2.5%B/g-C_3N_4.

Sample	S_{BET} (m^2/g)	Average pore size (nm)	Pore volume (cm^3/g)
pure g-C_3N_4	41	14.8	0.15
1%B/g-C_3N_4	62	11.2	0.17
2.5%B/g-C_3N_4	80	16.4	0.33

Table S3. The calculated adsorption energy of CO on pure g-C_3N_4 and B-doped g-C_3N_4.

**Samples**	pure g-C_3N_4	B-doped g-C_3N_4
**Adsorption energy (eV)**	0.36	-2.72

References

[1]. G. Kresse, J. Hafner, Phys. Rev. B **1994**, *49*, 14251

[2]. P. E. Blöchl, Phys. Rev. B **1994**, *50*, 17953

[3]. J. P. Perdew, K. Burke, M. Ernzerhof, Phys. Rev. Lett. **1996**, *77*, 3865

[4]. J. Heyd, G. E. Scuseria, M. Ernzerhof, J. Chem. Phys. **2003**, *118*, 8207.

[5]. J. Paier, M. Marsman, K. Hummer, G. Kresse, I. C. Gerber, J. G Ángyán, J. Chem. Phys. **2006**, *124*, 154709.

[6]. J. Heyd, G. E. Scuseria, M. Ernzerhof, J. Chem. Phys. **2006**, *124*, 219906.

[7]. S. Grimme, J. Comput. Chem. **2006**, *27*, 1787.

[8]. J. K. Nørskov, J. Rossmeisl, A. Logadottir, L. Lindqvist, J. Phys. Chem. B, **2004**, *108*, 17886.

[9]. J. Fu, B. Zhu, C. Jiang, B. Cheng, W. You, J. Yu, Small **2017**, *13*, 1603938.

[10].T. Di, J. Zhang, B. Cheng, J. Yu, J. Xu, Sci. China Chem. **2018**, *61*, 344.

[11].J. Yu, K. Wang, W. Xiao, B. Cheng, Phys. Chem. Chem. Phys. **2014**, *16*, 11492.
